# Accurate detection of subclonal single nucleotide variants in whole genome amplified and pooled cancer samples using HaloPlex target enrichment

**DOI:** 10.1186/1471-2164-14-856

**Published:** 2013-12-05

**Authors:** Eva C Berglund, Carl Mårten Lindqvist, Shahina Hayat, Elin Övernäs, Niklas Henriksson, Jessica Nordlund, Per Wahlberg, Erik Forestier, Gudmar Lönnerholm, Ann-Christine Syvänen

**Affiliations:** Department of Medical Sciences, Molecular Medicine and Science for Life Laboratory, Uppsala University, Uppsala, Sweden; Department of Medical Biosciences, University of Umeå, Umeå, Sweden; Department of Women’s and Children’s Health, Pediatric Oncology, Uppsala University, Uppsala, Sweden; For the Nordic Society of Pediatric Hematology and Oncology (NOPHO, Kragujevac, Sweden

**Keywords:** Target enrichment, HaloPlex, Non-indexed pooling, Whole genome amplification, Single nucleotide variant, Deep sequencing

## Abstract

**Background:**

Target enrichment and resequencing is a widely used approach for identification of cancer genes and genetic variants associated with diseases. Although cost effective compared to whole genome sequencing, analysis of many samples constitutes a significant cost, which could be reduced by pooling samples before capture. Another limitation to the number of cancer samples that can be analyzed is often the amount of available tumor DNA. We evaluated the performance of whole genome amplified DNA and the power to detect subclonal somatic single nucleotide variants in non-indexed pools of cancer samples using the HaloPlex technology for target enrichment and next generation sequencing.

**Results:**

We captured a set of 1528 putative somatic single nucleotide variants and germline SNPs, which were identified by whole genome sequencing, with the HaloPlex technology and sequenced to a depth of 792–1752. We found that the allele fractions of the analyzed variants are well preserved during whole genome amplification and that capture specificity or variant calling is not affected. We detected a large majority of the known single nucleotide variants present uniquely in one sample with allele fractions as low as 0.1 in non-indexed pools of up to ten samples. We also identified and experimentally validated six novel variants in the samples included in the pools.

**Conclusion:**

Our work demonstrates that whole genome amplified DNA can be used for target enrichment equally well as genomic DNA and that accurate variant detection is possible in non-indexed pools of cancer samples. These findings show that analysis of a large number of samples is feasible at low cost, even when only small amounts of DNA is available, and thereby significantly increases the chances of indentifying recurrent mutations in cancer samples.

**Electronic supplementary material:**

The online version of this article (doi:10.1186/1471-2164-14-856) contains supplementary material, which is available to authorized users.

## Background

During the past decade, next generation sequencing (NGS) technologies have revolutionized the field of human genetics. Since the first draft of the human genome was published in 2001 [1, 2], a multitude of personal genomes have been sequenced [3]. Large-scale efforts of whole genome sequencing (WGS) of human samples have mainly focused on population-based studies [4] and cancer genomes [5, 6]. For most research groups, WGS of many samples remains a costly endeavor, and targeted capture followed by sequencing of selected genomic regions of interest provides an attractive, cost-effective alternative. Target capture of custom designed regions and exome sequencing has allowed identification of causal variants in several Mendelian disorders [7], variants associated with complex diseases [8], and recurrently mutated cancer genes [9].

Target capture technologies can be categorized into methods based on PCR amplification, hybrid capture, or selective circularization [10]. The HaloPlex technology is a selective circularization-based method which is a further development of the principle of selector probes [11–13]. In the HaloPlex technology, genomic DNA is fragmented by restriction enzyme digestion and circularized by hybridization to probes whose ends are complementary to the target fragments. Compared to hybrid capture methods, the HaloPlex system requires smaller amounts of starting DNA, has higher specificity (fraction of sequence reads in the region of interest), and provides more even genome coverage [10]. The sample preparation is less laborious because incorporation of sequencing adapters during capture obviates the need for a separate library preparation step. However, a maximum of 5 Mb of custom designed regions can be enriched with current protocols, which is little compared to for example 24 Mb for SureSelect hybrid capture.

To further decrease costs, samples can be pooled prior to capture. Indexing of samples before pooling allows posterior identification of the sample where a variant occurred, and results in more accurate estimation of allele frequencies. However, the presence of an additional index tag may complicate experimental procedures, decrease capture specificity, and result in a substantial proportion of reads with an inappropriate index tag [14, 15]. Indexed pooling is feasible in hybrid capture methods, where the sequencing library is prepared before capture, but not for the current HaloPlex protocol, where the capture is performed on genomic DNA. Pooling without indexing can be done at the level of genomic DNA, and is thus an efficient approach in terms of reagent costs and experimental work load. While non-indexed pooling has not yet been used with the HaloPlex technology, several studies have evaluated the performance of non-indexed pools using hybrid capture. These studies have yielded conflicting results, with some observing accurate SNP calling and determination of allele frequencies [15, 16], while others observe poor allele frequency estimates or failure to validate SNPs with minor allele frequencies below 25% [17, 18].

Previous studies of non-indexed pooling aimed at establishing methods for detecting rare variants in complex phenotypes, using HapMap cell lines or other non-tumor samples. To our knowledge, pooling of cancer samples for identification of somatic single nucleotide variants (SNVs) has not been evaluated to date. In contrast to germline SNPs, most somatic SNVs are not expected to be recurrent in the population, nor in other individuals with the same type of cancer. Additionally, cancer samples often consist of multiple tumor subclones in mixtures with normal cells, and the proportion of sequence reads containing an SNV can therefore be very low in pools of cancer samples. Another consideration when working with tumor samples is that DNA resources are often limited. Whole genome amplification (WGA) by multiple displacement amplification is a standard method to increase DNA quantity. Evaluation of whole genome amplified DNA (wgaDNA) for genotyping purposes has shown that the allele fractions in the original sample are retained provided that a sufficient amount of genomic DNA is used [19, 20]. The use of wgaDNA in WGS can result in uneven coverage and introduce false positive inversions [21], and in hybrid capture it has been associated with accurate SNP detection but a slightly decreased capture specificity [22]. However, it has not been properly investigated using NGS how well the allele fractions of somatic SNVs are preserved during WGA.

In this study, we investigated the power to detect somatic SNVs in non-indexed pools of up to ten cancer samples and the effect of WGA on capture specificity and allele fractions using HaloPlex target enrichment. We analyzed 1528 candidate SNVs and SNPs identified by WGS of matched cancer and normal samples from two patients with acute lymphoblastic leukemia (ALL).

## Results and discussion

### Design of HaloPlex target enrichment experiment

For evaluation of the performance of whole genome amplified DNA (wgaDNA) and non-indexed pooling in HaloPlex target enrichment, we selected 1541 candidate single nucleotide variants (SNVs) detected during whole genome sequencing (WGS) of two patients with acute lymphoblastic leukemia (ALL). Thirty of the SNVs were previously validated as somatic by PCR and Sanger sequencing, and one had been shown to be a false positive. No indels were included. The candidate SNVs selected for evaluation of the HaloPlex target enrichment system included 749 SNVs from patient 1 and 794 from patient 2. Two of the SNVs overlapped between the patients. We also selected 20 germline SNPs that were heterozygous in both patients. For each of these 1561 variants (1541 candidate SNVs and 20 SNPs), we defined a target region of 3 bp, including one base upstream and one base downstream of each variant. In addition, we selected the exons of 37 genes and five custom regions with a size ranging from 33 to 263 bp. The total number of target regions was 2431, and together they spanned 147 kb. The design obtained from Agilent had a total size of 798 kb and covered 99.6% of the region of interest. A total of 1528 (97.9%) of the SNVs and SNPs were covered by the design, including 1509 candidate SNVs (726 in patient 1 and 785 in patient 2, including the two overlapping SNVs) and 19 of the 20 germline SNPs. The failure to completely cover a region of interest can be attributed to repeated regions in the flanking sequences, lack of restriction fragments of appropriate size, or too large fragments relative to the read length leading to partial sequencing of fragments.

A total of twelve samples or pools were subjected to target capture using the HaloPlex system (Table 1). First, for validation of putative SNVs detected in WGS data, we included genomic DNA (gDNA) from cancer and normal samples from the two whole genome sequenced ALL patients, here called ALL1, ALL2, Normal1, and Normal2. Second, to evaluate the effect of whole genome amplification, we included wgaDNA from the ALL1 and ALL2 samples. Third, for evaluation of non-indexed pooling, we prepared three pools of two, five or ten ALL samples. Each pool contained either the ALL1 or the ALL2 sample, and the additional samples were present in only one pool each. The pools were prepared in replicate by an independent pooling procedure.

**Table 1:** **Samples and sequence data statistics**

Sample or pool^a^	Average sequence depth		Cumulative depth at variants (%)		Mapped reads (%)^d^	Mapped on target (%)^e^
	Design^b^	Variants^c^	≥1x	≥30x		
ALL1_gDNA	1385	1940	99.4	96.5	92.4	67.1
Normal1_gDNA	1280	1588	99.6	96.2	91.4	60.8
ALL2_gDNA	1466	1791	99.4	95.7	92.2	64.7
Normal2_gDNA	792	1008	99.2	95.2	91.8	64.0
ALL1_wgaDNA	1564	1831	99.2	94.2	92.6	73.5
ALL2_wgaDNA	1569	1706	99.1	94.0	92.5	73.0
ALL1_pool2	1752	2254	99.7	97.4	91.8	67.7
ALL2_pool5	1502	1710	99.4	96.9	93.1	68.8
ALL1_pool10	1044	1445	99.6	96.8	89.4	63.0
ALL1_pool2_rep	1150	1614	99.4	95.9	84.5	42.4
ALL2_pool5_rep	1021	1503	99.3	91.6	85.5	49.9
ALL1_pool10_rep	1386	1837	99.5	96.3	85.9	58.5

^a^ Pools are named with the whole genome sequenced ALL sample included and the total number of samples in the pool.

^a^ Average sequence depth in the complete region covered by the HaloPlex design.

^c^ Average sequence depth at the 1528 candidate SNVs and SNPs covered by the HaloPlex design.

^d^ Percentage of sequence reads that map to the human genome.

^e^ Percentage of the sequence reads mapping to the genome that map to the regions covered by the HaloPlex design.

### Sequence data and allele fractions

Between 84.5% and 93.1% of the raw sequence reads mapped to the human genome, and 42.4-73.5% of these mapped to the target region (Table 1). The average sequence depth ranged from 792 to 1752 in the region covered by the HaloPlex design, and from 1008 to 2254 at the 1528 SNVs and SNPs covered by the capture design (Table 1). Virtually all SNVs and SNPs were covered by at least one sequence read, and 91.6-97.4% of the variants were covered at a sequence depth of at least 30 (Table 1). To determine the accuracy of the allele fractions in the HaloPlex sequence data we utilized the 19 heterozygous germline SNPs, which are expected to have an allele fraction of 0.5 in individual samples from both cancer and normal cells. The allele fractions of these SNPs deviated from the expected 0.5 by an average of 0.064 in the HaloPlex data, compared to 0.135 in the WGS data, demonstrating that the increased sequencing depth in the HaloPlex data is associated with increased precision of allele fractions (Additional file 1: Figure S1).

### Somatic SNVs

**Figure 1:**
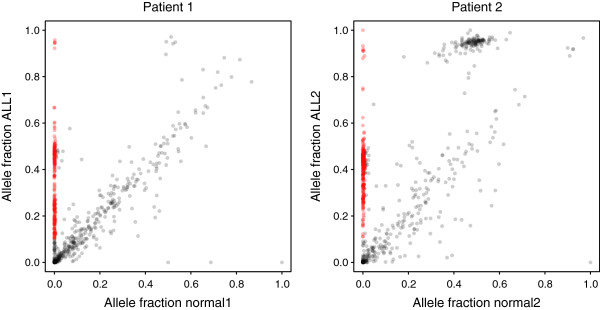
**Allele fractions for putative single nucleotide variants (SNVs) in ALL and normal samples.** The allele fractions observed in HaloPlex sequence data for 1509 candidate SNVs in ALL and normal samples. Only data from libraries derived from genomic DNA are shown. SNVs classified as somatic are shown in red. Candidate SNVs that follow the x = y line represent putative germline SNPs that escaped detection in the normal sample during WGS or alignment artifacts. The cluster with allele fractions close to 0 in both ALL and normal samples represents likely false positive SNV calls. Most of the candidate SNVs with allele fractions close to 1 in the ALL sample and around 0.5 in the normal sample in patient 2 are located in a large region of somatic loss of heterozygosity.

Accurate identification of somatic SNVs in WGS data is challenging. Different SNV callers often yield different results and large numbers of false positive calls [23], and experimental validation is often required. Target capture and deep sequencing is a well established method for validation of large numbers of putative SNVs detected in WGS data [24–26]. Here, we used the HaloPlex deep-sequencing data to classify our candidate SNVs as somatic or “non-validated”. At the sites where a putative SNV was detected in the WGS data, we determined the allele fractions in the HaloPlex data, and classified an SNV as somatic if it had an allele fraction ≥0.1 in the gDNA ALL sample and <0.01 in the matched normal sample. We also required a sequence depth ≥30 in both samples in the sequencing data from HaloPlex enrichment. This resulted in 227 and 305 SNVs classified as somatic in ALL1 and ALL2, respectively, corresponding to approximately one third of the candidate SNVs called from WGS data (Figure 1). As a measure of confidence, all of the 30 previously validated somatic SNVs were classified as somatic in the HaloPlex sequence data. The candidate SNV that had previously been shown to be a false positive by Sanger sequencing had no supporting reads in the HaloPlex data.

The candidate SNVs that were not somatic displayed three distinctive patterns. First, many candidate SNVs align along the diagonal, with similar allele fractions in the ALL and normal samples. These SNVs could possibly be germline SNPs that escaped detection in the normal sample in the WGS data. This hypothesis is supported by the observation that they have significantly lower coverage in the WGS data in the normal sample than the validated somatic SNVs (Additional file 1: Figure S2). We also observed significantly lower confidence scores from SNV calling in WGS, as measured by the somatic score from SomaticSniper [27], in this group of candidate SNVs (Additional file 1: Figure S2). However, germline SNPs would be expected to have an allele fraction close to 0.5. Analysis of mappability scores and overlaps with segmental duplications suggests that some of the putatively germline SNPs with deviating allele fractions might be located in repeated regions of the genome that had escaped our filters (data not shown). Manual inspection of sequence alignments suggests that other candidates in this category are false positive SNVs caused by alignment artifacts. Second, there is a cluster of non-validated SNVs in the lower left corner in Figure 1. Similarly to the previous category, these candidate SNVs display low coverage in the normal sample in WGS data and low somatic scores (Additional file 1: Figure S2). This observation suggests that the failure to detect these SNVs in the HaloPlex data is not caused by allelic dropout during HaloPlex enrichment, but rather by sequencing errors or alignment artifacts in combination with low coverage leading to false positive SNV calls in the WGS data. Third, ALL2 shows a cluster of candidate SNVs with allele fractions around 0.5 in the normal sample and close to 1 in the ALL sample. Most of these variants are located in a previously known large chromosomal region of somatic loss of heterozygosity.

To further verify that our criteria for classifying a putative SNV as somatic are valid, ten somatic SNVs (five from each patient), with allele fractions between 0.2 and 0.8, were randomly selected for PCR and Sanger sequencing. All ten SNVs were successfully validated as being somatic. This result, along with the different properties of somatic and non-validated SNVs, shows that we can accurately validate somatic SNVs using HaloPlex enrichment and deep sequencing.

### Comparison of wgaDNA and gDNA

**Figure 2:**
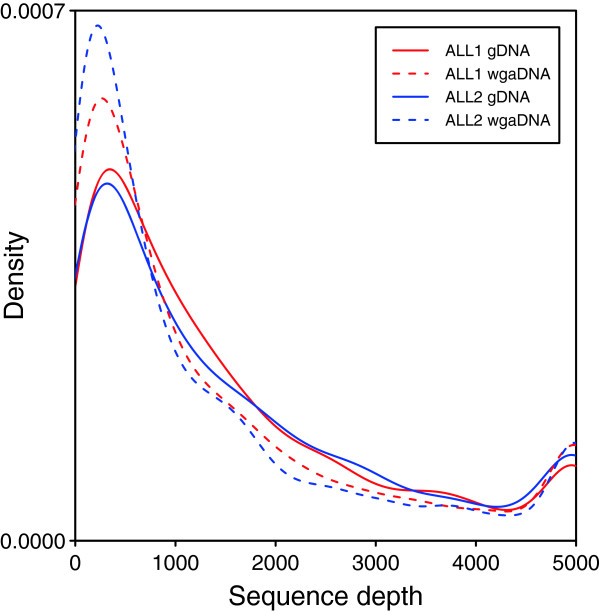
**Sequence depth variation in genomic DNA (gDNA) and whole genome amplified DNA (wgaDNA) samples.** Density plot showing the variation in HaloPlex sequence depth of 1509 candidate single nucleotide variants (SNVs) and 19 germline SNPs in gDNA and wgaDNA samples. The sequence depth is more uneven in the wgaDNA samples, which display relatively low or high coverage at more sites. To increase clarity, sites with a depth > 5000 are shown at 5000 and the x-axis has been cut at 0 and 5000.

To evaluate whether alleles were lost or allele fractions were altered during whole genome amplification, we compared the results from gDNA and wgaDNA in the samples ALL1 and ALL2. The percentage of reads on target was slightly higher for the wgaDNA samples than for the gDNA samples, showing that whole genome amplification does not have a negative effect on capture specificity (Table 1). A possible explanation for the higher specificity of wgaDNA samples would be that genomic regions that are easier to capture with the HaloPlex method are preferentially amplified during the WGA process. However, since the number of experiments is limited, the observed pattern could also be caused by random variation. A slightly smaller number of variants had a sequence depth ≥1 and ≥30 in the wgaDNA samples (Table 1). Further investigation showed that the coverage in wgaDNA samples is more uneven, with more sites covered by a relatively low or high number of reads (Figure 2). There was no correlation between the amount of input DNA, which ranged from 200 to 1000 ng in the pools, and the evenness of the coverage (Additional file 1: Figure S3), suggesting that using more of the wgaDNA would not have yielded more even coverage. These results indicate that in order to achieve adequate coverage of the target regions, it is important to sequence wgaDNA samples at sufficient depth. The number of SNVs classified as somatic was similar in the gDNA and wgaDNA samples (Table 2). A total of 29 SNVs were classified as somatic in only one of the gDNA and wgaDNA experiments using the same sample (Table 2). Five of them can be explained by lack of coverage in the wgaDNA sample. Manual inspection of the sequence data at the remaining SNVs suggested that the inconsistencies were due to alignment artifacts or allele fractions close to the cutoff, rather than allelic dropout during whole genome amplification.

**Table 2:** **Detection of single nucleotide variants and error rates in genomic DNA and whole genome amplified DNA**

Sample	Somatic SNVs^a^	Error^b^
ALL1_gDNA	227 (13)	0.036
ALL1_wgaDNA	223 (9)	0.020
ALL2_gDNA	305 (5)	0.038
ALL2_wgaDNA	302 (2)	0.031

^a^ Within parenthesis is the number of candidate SNVs that were not classified as somatic in the corresponding gDNA or wgaDNA experiment from the same original sample.

^b^ Average absolute difference from 0.5 for 19 germline SNPs.

**Figure 3:**
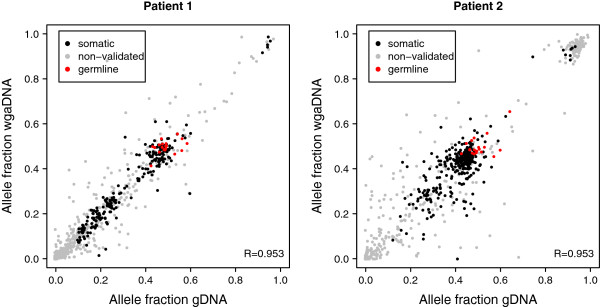
**Correlation between allele fractions in genomic DNA (gDNA) and whole genome amplified DNA (wgaDNA).** Correlation of the allele fractions of candidate single nucleotide variants (SNVs) and germline SNPs determined in experiments using gDNA and wgaDNA from the same original DNA sample. Only variants with a HaloPlex sequence depth ≥30 in both gDNA and wgaDNA are shown (n = 1439). SNVs classified as somatic are shown in black, non-validated candidate SNVs in grey, and germline SNPs in red.

For analysis of how well allele fractions are preserved during whole genome amplification, we used all candidate SNVs detected in the WGS data as well as the germline SNPs, with a sequence depth ≥30 in both gDNA and wgaDNA experiments (n = 1439 variants). This was because allele fractions are expected to be preserved for all variants. In addition, including all variants allows investigation of the preservation of the allele fractions across the complete range of allele fractions (*i.e.*, 0–1). Cancer samples with variants with varying allele fractions are therefore particularly well suited to assess how well allele fractions are preserved during whole genome amplification. The error in allele fraction for the 19 germline SNPs was slightly smaller in wgaDNA than in gDNA, suggesting that the uneven coverage in wgaDNA samples does not affect the allele fractions (Table 2). We observed a high correlation between allele fractions obtained in data from gDNA and wgaDNA experiments, with Pearson correlation coefficients of 0.953 in both patients (Figure 3). The reason why the two patients have the same correlation value, although there appears to be more variation in ALL2, is that the cluster of data points in the upper right corner has a major effect on the correlation. To better estimate the variation in the datasets, we also calculated the root mean square error (RMSE). This value was 0.066 and 0.093 for ALL1 and ALL2, respectively. In addition to demonstrating that wgaDNA provides a good substitute for gDNA when DNA resources are limited, our results highlight the technical reproducibility of HaloPlex enrichment.

### Allele fractions in pooled DNA samples

**Figure 4:**
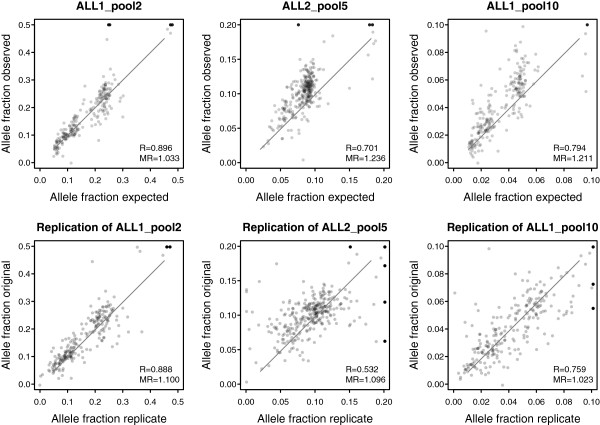
**Correlation between expected and observed allele fractions in pools and comparison of replicated pools.** The top panel shows the correlation between expected and observed allele fractions in the three pools, where the expected value is calculated by dividing the allele fraction observed in the ALL1 or ALL2 genomic DNA (gDNA) sample with the number of samples in the pool. Only single nucleotide variants (SNVs) classified as somatic in the ALL1 (n = 227) or ALL2 (n = 305) gDNA samples are shown. The bottom panel shows the correlation between allele fractions in the replicated pools. The x- and y-axes are cut at 0.5, 0.2 and 0.1 for the pools with two, five and ten samples, respectively. SNVs with an observed allele fraction greater than this are shown in black at the cutoff value. The MR value is the median ratio between all x- and y-values in each plot. It represents how close the two distributions are to each other, with a value of 1 indicating similar distributions.

For evaluation of variant detection and how well allele fractions are preserved in pools, we focused on the somatic SNVs (n = 227 in ALL1 and n = 305 in ALL2). These variants are likely to be unique to the whole genome sequenced sample, and therefore an expected allele fraction for each variant can be calculated by dividing the allele fraction in the individual gDNA sample with the number of samples in the pool. In contrast, germline SNPs are more likely to be present in some of the additional samples included in the pool, which hinders the use of these variants to determine the precision of the method. A comparison between observed and expected allele fractions showed good correlation in all pools (Figure 4, top panel). The allele fractions in the pools containing ALL1 displayed higher levels of correlation than the pool containing ALL2. A possible explanation is that ALL1 contains clear leukemic subclones with approximately half of the SNVs having an allele fraction around 0.2 and the remaining having an allele fraction around 0.45 (Figures 3 and 4). In contrast, the majority of somatic SNVs in ALL2 have an allele fraction around 0.45, and this lower variability decreases the correlation. In ALL2_pool5 and ALL1_pool10, the observed allele fractions were on average higher than the expected. We quantified the deviation between observed and expected allele fractions by calculating the median ratio of x- and y-values in each plot. This ratio would be close to 1 if the data is evenly distributed around the x-y line, as in the case of ALL1_pool2. For ALL2_pool5 and ALL1_pool10 the ratio was around 1.2. This result suggests that approximately 25% and 12%, respectively, of these pools consisted of the ALL2 and ALL1 samples, compared to the expected 20% and 10%.

When comparing the pool replicates against each other, the correlation between observed allele fractions was somewhat lower than the correlation between observed and expected values in the “original” pool (Figure 4, bottom panel). This was not unexpected, since the variance is likely to increase in pools compared to individual reactions due to additional experimental factors and decreased sequencing depth per sample. Thus, in the replicate plots we compare two high-variance experiments, while in the observed-expected plots the expected values are obtained from a low-variance experiment. Despite increased variance, the median ratio between allele fractions in the replicates was close to 1, suggesting that pooling is reproducible and that the skewed distributions in ALL2_pool5 and ALL1_pool10 may be caused by inaccurate DNA quantification of the individual samples in the pools or by variations from pipetting small volumes of DNA samples during pooling.

### Variant detection in pooled DNA samples

Since the genome sequence was unknown for all samples in the pools, except ALL1 and ALL2, we could not use the entire target region to estimate the accuracy of the SNV calling. Instead we focused on the sites for which we have genetic information about somatic SNVs in ALL1 and ALL2. Since we used an allele fraction cutoff of 0.1 to call a somatic SNV in the HaloPlex data, the lowest expected allele fraction in a pool is 0.1 divided by the number of samples in the pool. To allow for inaccuracy of the DNA quantification or pipetting, and random variation of sequenced molecules, a somatic SNV was considered to be detected in a pool if the allele fraction at this site was at least half the lowest expected value. To estimate the number of true negatives and false positives, we reasoned that the somatic SNVs are rare, so that somatic SNVs detected in ALL1 would not be present in pools containing ALL2 and vice versa. This analysis showed that the vast majority of the known SNVs present in a single sample can be reliably detected in the pools, with the number of false negatives ranging from 1 to 7 and the number of false positives ranging from 1 to 14 (Table 3). As expected, more false positives were observed in pools of many samples, however, the false discovery rate was below 6% for all pools (Table 3). To investigate whether the higher observed than expected allele fractions in ALL2_pool5 and ALL1_pool10 cause an upward bias in the number of detected SNVs, we adjusted the observed allele fractions by dividing them by the median ratio between observed and expected values. SNV calling using the adjusted allele fractions showed that only one of all the SNVs that were detected in the original analysis remained undetected after scaling. Thus, the overrepresentation of ALL1 and ALL2 in these pools does not influence the accuracy estimate. A closer inspection of the SNVs that remained undetected in pools revealed that the majority had a low expected allele fraction and a relatively low sequence depth (Additional file 1: Figure S4), suggesting that deeper sequencing would allow detection of most SNVs. We also found that the SNVs that remained undetected in at least one pool had a significantly lower sequence depth in the individual ALL1 and ALL2 samples compared to the remaining SNVs, suggesting that they may be located in genomic regions that are difficult to enrich.

**Table 3:** **Accuracy of single nucleotide variant detection in non-indexed pools**

Sample	TP^a^	TN^a^	FP^a^	FN^a^	FDR (%)^b^	Novel^c^
ALL1_pool2	224	304	1	3	0.4	0
ALL2_pool5	304	227	0	1	0	3
ALL1_pool10	225	296	9	2	3.8	4
ALL1_pool2_rep	224	304	1	3	0.4	0
ALL2_pool5_rep	298	222	5	7	1.7	2
ALL1_pool10_rep	223	291	14	4	5.9	4

^a^ TP: true positives; TN: true negatives; FP: false positives; FN: false negatives.

^b^ FDR: false discovery rate, calculated as FP/(TP + FP).

^c^ Putative novel SNVs called in the previously uncharacterized samples included in the pools. One of the variants called in ALL2_pool5 was not called in the corresponding replicated pool (ALL2_pool5_rep). All other novel variants were identical between replicates.

To investigate whether novel variants could be discovered in the previously uncharacterized samples in the pools, we performed a *de novo* SNV calling in the entire region of interest (147 kb) by calculating the allele fraction at all sites in this region. To filter out putatively germline SNPs and false positive calls, we set the expected allele fraction for a somatic SNV present in a single sample in a pool to 0.5 divided by the number of samples included in the pool and we selected only variants with an allele fraction between half and twice the expected value. We also filtered against dbSNP and the other experiments included in the study (see Methods for details). After applying these filters, we identified six high-confidence candidate somatic SNVs that were called in both replicates of the same pool (Table 3). One additional SNV was called in pool ALL2_pool5 but not in the replicate of this pool (Table 3). Manual inspection of this variant suggested that it was a false positive call caused by alignment artifacts. We performed PCR and Sanger sequencing of each of the samples included in the pools where the six high-confidence SNVs were identified. In each case, we were able to show that it was a true variant and in which DNA sample it was present. Pooling of DNA samples before capture thus allows accurate SNV detection in many samples at low reagent cost, but at the expense of losing the information of in which sample novel variants are detected, unless experimental validation is performed. If the variants are expected to be so rare that they occur in only one sample, this limitation can potentially be circumvented by using a pool design where each sample is present in two different pools and no other sample is present in both pools [28]. In most studies, cancer samples are compared to matched normal controls. Non-indexed pooling could be particularly useful for the controls, since variants present in the normal population are not believed to be cancer mutations, even if the presence of the variant in a matched normal sample has not been confirmed.

## Conclusions

In this work, we analyzed 1528 putative somatic SNVs and germline SNPs with the HaloPlex target enrichment technology and NGS to evaluate the performance of whole genome amplified DNA and the accuracy of SNV detection in non-indexed pools of cancer samples. We selected the HaloPlex technology since it is a novel, fast, and specific method suitable for targeted sequencing of relatively small regions in many samples. We found that the allele fractions of the analyzed variants are well preserved during whole genome amplification, with correlation coefficients of 0.953 between gDNA and wgaDNA samples, and that WGA does not negatively affect capture specificity or variant calling. The possibility of using wgaDNA is particularly important in cancer research, since the amount of available DNA is often a major limitation when working with primary human cancer samples. Furthermore, we show that using a sequence depth of 792–1752, SNVs present in a single sample with allele fractions as low as 0.1 can be reliably detected in non-indexed pools of up to ten samples. We also identified and experimentally validated six novel variants in the samples that were included in these pools. Our results are important, since they show that analysis of a large number of samples, including samples where limited amounts of DNA have previously been prohibitive, is possible at low cost. Since analysis of many samples significantly increases the chances of finding recurrent cancer genes, our results have great potential to be beneficial for cancer research.

## Methods

### Samples

Sixteen bone marrow samples collected at diagnosis from patients with childhood acute lymphoblastic leukemia (ALL) were analyzed in this study (Additional file 1: Table S1). All patients were treated at Swedish centers according to the Nordic Society for Pediatric Haematology and Oncology (NOPHO) 1992 and 2000 ALL protocols [29]. The study was approved by the Regional Ethical Review Board in Uppsala, Sweden. The study was conducted according to the guidelines of the Declaration of Helsinki, and all patients and/or guardians provided written or oral informed consent.

We have previously performed whole genome sequencing of two of the ALL samples included in the study (ALL1 and ALL2), along with matched normal samples from the same patients (Lindqvist *et al.*, manuscript in preparation). Both patients responded well to therapy, and at examination after cessation of therapy 2-2½ years after diagnosis they were found to be in first continuous complete remission (CCR 1), with <0.01% blast cells in the bone marrow according to PCR. The normal blood samples were collected 2½-3 years later, when the patients were still in CCR 1 with normal hematological parameters. The patients are today clinically well in CCR 1 another 2½-3 years later. Thus, there is good evidence that the normal blood samples did not contain any leukemic cells. The proportion of leukemic cells in the cancer samples was estimated to be >90% by light microscopy in May-Grünwald-Giemsa-stained cytocentrifuge preparations.

For whole genome sequencing, on average 138 Gb paired-end sequence data was generated for each sample using the HiSeq2000 or GAIIx instruments (Illumina). Sequence reads were trimmed from the 3’ end and aligned to the human reference genome (version hg19) using BWA version 0.5.9 [30] with default parameters. Read realignment and base quality recalibration was performed using GATK version 1.0.5909 [31]. Read realignment was performed around candidate indels identified during the run and indels previously called in the data using VarScan [32]. During base quality recalibration, dbSNP132 and the BAQ option was used. PCR duplicates and read pairs where at least one read fulfilled any of the following criteria were excluded: trimmed to <25 bp, >3 mismatches or MAPQ <30. Somatic SNVs were predicted with MuTect version 1.0.27200 [33] and SomaticSniper version 1.0.0 [27] with default parameters. MuTect SNVs labeled REJECT and SomaticSniper SNVs with somatic score <40 were discarded. In addition, SNVs with an allele fraction <0.2, SNVs present in dbSNP135, and SNVs overlapping a repeated region present in the tracks “rmsk” or “simpleRepeats” from the UCSC table browser were excluded from further analysis.

### Target capture and sequencing

Capture of the target regions was performed with reagents from a custom design HaloPlex Target Enrichment kit 1-500 kb (Agilent, USA), according to the HaloPlex Target Enrichment System-Fast Protocol Version B. Briefly, the protocol consists of the following four steps: 1) Digestion of genomic DNA in eight different restriction reactions. 2) Hybridization of restricted fragments to probes whose ends are complementary to the target fragments. During hybridization, fragments are circularized and sequencing motifs including index sequences are incorporated. 3) Capture of target DNA using streptavidin beads and ligation of circularized fragments. 4) PCR amplification of captured target libraries. Recent revisions of the protocol are listed in Additional file 2.

Whole genome amplification was performed using 50 ng of genomic DNA with reagents from the Repli-g midi kit (Qiagen), according to the manufacturer’s instructions. 200 ng of DNA was used for capture reactions containing a single sample. 100 ng of DNA from each sample was used for pooling and all pooled DNA was used in the capture reaction. Thus, the amount of input DNA ranged from 200–1000 ng in the capture experiments. Prior to pooling, DNA was quantified using a Qubit Fluorometer (Invitrogen). Paired-end sequencing (100 bp reads) of all samples was performed in a single lane on a HiSeq2000 instrument (Illumina, USA). A HiSeq Paired End Cluster Generation Kit was used to generate the clusters and a TruSeq SBS Kit v3 was used for sequencing. Image analysis and base calling was performed using the Illumina RTA software version 1.13.48.

### Data analysis

Sequence reads were trimmed to remove Illumina adapter sequences with CutAdapt version 1.1 [34] and aligned to the human reference genome (version hg19) with MOSAIK version 2.1.33 with default parameters. Realignment and recalibration of base quality scores using dbSNP137 was performed with GATK version 1.0.5909 [31]. Read realignment was performed around candidate indels identified during the run, and SNPs and indels in dbSNP137 that were located in the regions covered by the HaloPlex design. Reads with MAPQ = 0 were discarded.

Allele fractions at sites with candidate SNVs detected in WGS data and germline SNPs were calculated with a custom Python script (publicly available at https://github.com/Molmed/Berglund-Lindqvist-2013). Variant calling was based on these allele fractions. In individual samples, a candidate SNV was classified as somatic if fulfilling the following criteria: allele fraction ≥0.1 in the gDNA ALL sample, allele fraction <0.01 in the matched normal sample, and HaloPlex sequence depth ≥30 in both samples. In pools, we considered a validated somatic SNV to be detected if the allele fraction was ≥0.05 divided by the number of samples in the pool.

For *de novo* SNV calling in pools, we investigated the allele fractions at every site in the 147 kb target region that had a sequence depth ≥30 per sample included in the pool. We only searched for variants in the unknown (*i.e.*, not whole genome sequenced) samples. We applied several criteria to filter out putative germline SNPs and false positive calls. First, we assumed that variants that are present in more than one of the samples in a pool are likely to be germline, and we focused on finding variants with an allele fraction suggesting that they are present in only one sample. We set the expected allele fraction for such variants to 0.5 divided by the number of samples in the pool. We excluded variants with an allele fraction less than half or more than twice the expected value. Second, we filtered out all variants present in dbSNP137. Third, we excluded variants that had an allele fraction >1% in any of the other experiments included in the study except the replicate experiment. This was to filter out germline variants that are not in dbSNP and putative false positive calls caused by alignment artifacts. Validation of putative novel SNVs was done by PCR amplification and Sanger sequencing of each of the samples included in the pools individually. Figures were generated using R version 3.0.1.

### PCR and Sanger sequencing

PCR primers were designed using Primer3Plus [35]. PCR was performed using a Smart Taq Hot Thermostable DNA Polymerase Set (Naxo, Estonia) for 35 cycles. Sanger sequencing was performed with an ABI3730XL instrument at the Genome Center in Uppsala, Sweden. The sequence traces were analyzed with the Sequencher software (Applied Biosystems).

## Electronic supplementary material

Additional file 1: Figure S1: The allele fractions and sequencing depths of 19 heterozygous germline SNPs in HaloPlex and whole genome sequencing data. **Figure S2.** Comparison of somatic and non-validated single nucleotide variants (SNVs) in terms of sequence depth for the normal sample in whole genome sequencing data and confidence score from SNV calling as measured by the somatic score from SomaticSniper. **Figure S3.** Comparison of the evenness of coverage, as estimated by the coefficient of variation (CV) and the Gini index, between experiments with different amounts of input DNA. **Figure S4.** The sequence depth and expected allele fraction of somatic SNVs that remained undetected in pools. **Table S1.** Clinical characteristics of ALL samples used in the study. (PDF 125 KB)

Additional file 2: **List of revisions of the HaloPlex target capture protocol between our experiment and the newest version of the protocol.** (PDF 405 KB)

## Abbreviations

SNP: Single nucleotide polymorphism
SNV: Single nucleotide variant
WGS: Whole genome sequencing
WGA: Whole genome amplification
wgaDNA: Whole genome amplified DNA
gDNA: Genomic DNA
NGS: Next generation sequencing.
