# Supplementary material for: Accurate detection of subclonal single nucleotide variants in whole genome amplified and pooled cancer samples using HaloPlex target enrichment
Source: BMC Genomics. 2013 Dec 5;14(1):856. doi: 10.1186/1471-2164-14-856 (PMC4046713; doi:10.1186/1471-2164-14-856)
Supplement: Supplementary file 1 — Additional file 1: Figure S1: The allele fractions and sequencing depths of 19 heterozygous germline SNPs in HaloPlex and whole genome sequencing data. Figure S2. Comparison of somatic and non-validated single nucleotide variants (SNVs) in terms of sequence depth for the normal sample in whole genome sequencing data and confidence score from SNV calling as measured by the somatic score from SomaticSniper. Figure S3. Comparison of the evenness of coverage, as estimated by the coefficient of variation (CV) and the Gini index, between experiments with different amounts of input DNA. Figure S4. The sequence depth and expected allele fraction of somatic SNVs that remained undetected in pools. Table S1. Clinical characteristics of ALL samples used in the study. (PDF 125 KB) [file 12864_2013_5568_MOESM1_ESM.pdf]

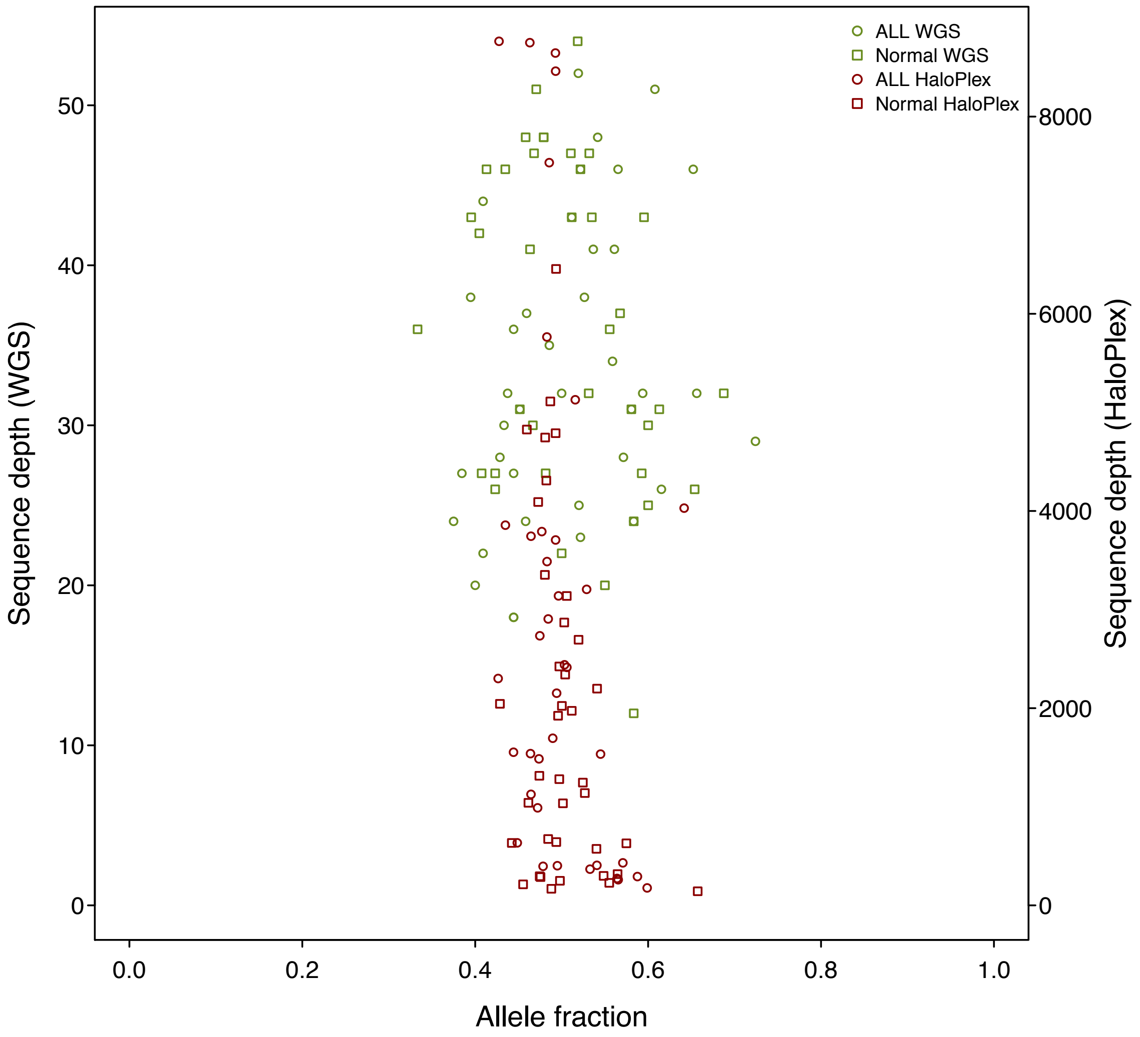

Figure S1. Allele fractions and sequencing depths of germline SNPs in HaloPlex and whole genome sequencing (WGS) data. The allele fractions of 19 heterozygous germline SNPs in HaloPlex and WGS data are shown for the four whole genome sequenced samples (ALL1, ALL2, Normal1, and Normal2). Only data from libraries derived from genomic DNA are shown. The left y-axis shows the sequence depth for the WGS data and the right y-axis shows the sequence depth for the HaloPlex data.

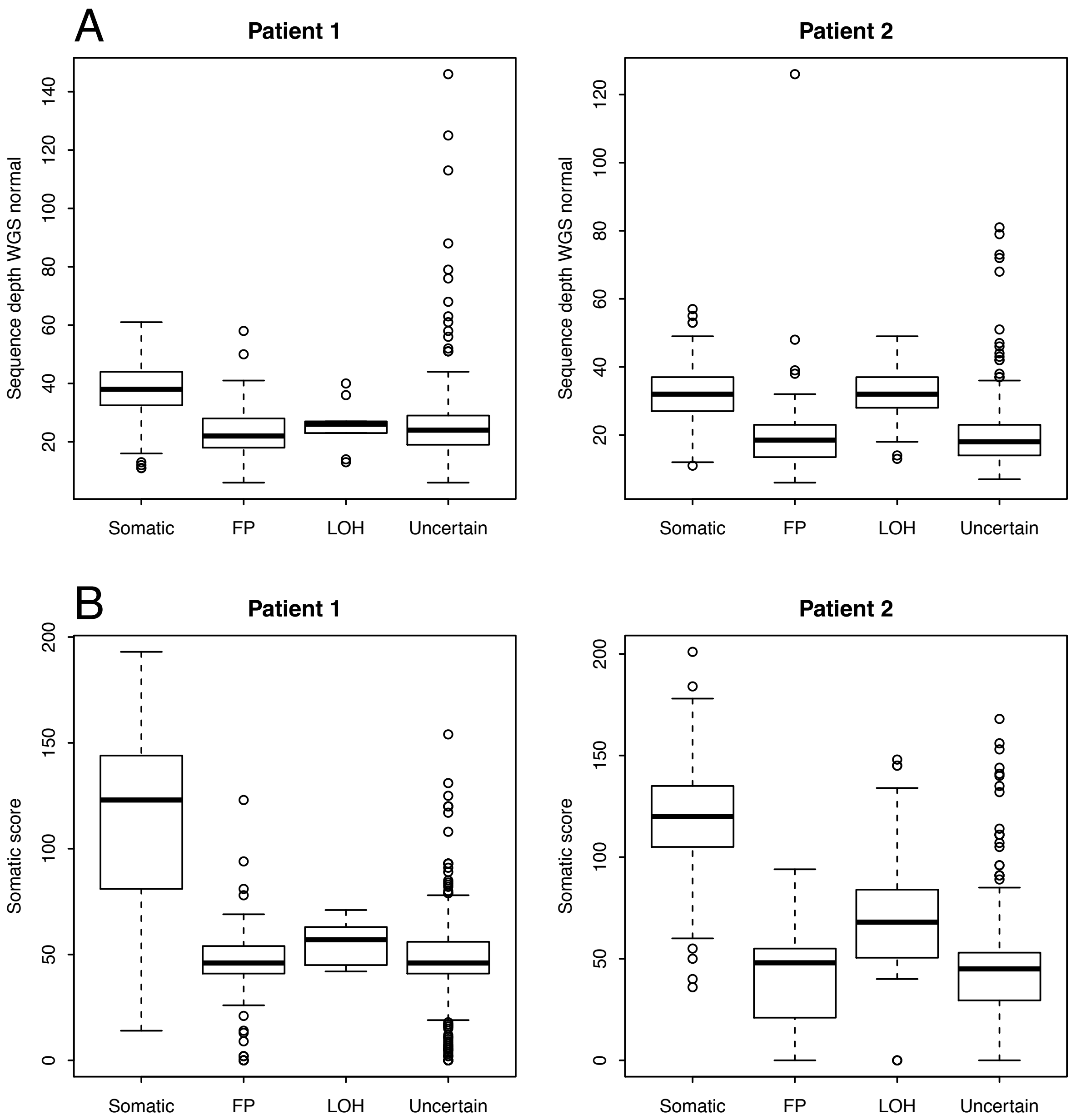

Figure S2. Characteristics of somatic and non-validated single nucleotide variants (SNVs). Comparison of somatic and non-validated SNVs in terms of A) sequence depth in the normal sample in whole genome sequencing (WGS) data, and B) confidence score from SNV calling in WGS data as measured by the somatic score from SomaticSniper. The definitions of subgroups of candidate SNVs predicted from WGS data are as follows: **Somatic**: allele fraction from HaloPlex data ≥0.1 in the ALL sample and <0.01 in the normal sample; Putative false positives (**FP**): allele fraction <0.01 in both the ALL sample and the normal sample; Loss of heterozygosity (**LOH**): allele fraction ≥0.8 in the ALL sample and between 0.1 and 0.9 in the normal sample; For classification of a candidate SNV as somatic, FP or LOH, we also required a sequence depth ≥30 in both the ALL and the normal sample. **Uncertain**: remaining candidate SNVs. The somatic SNVs have significantly higher somatic score than all groups of nonvalidated SNVs, and significantly higher sequence depth in the normal sample in WGS data than the SNVs in the FP and Uncertain groups.

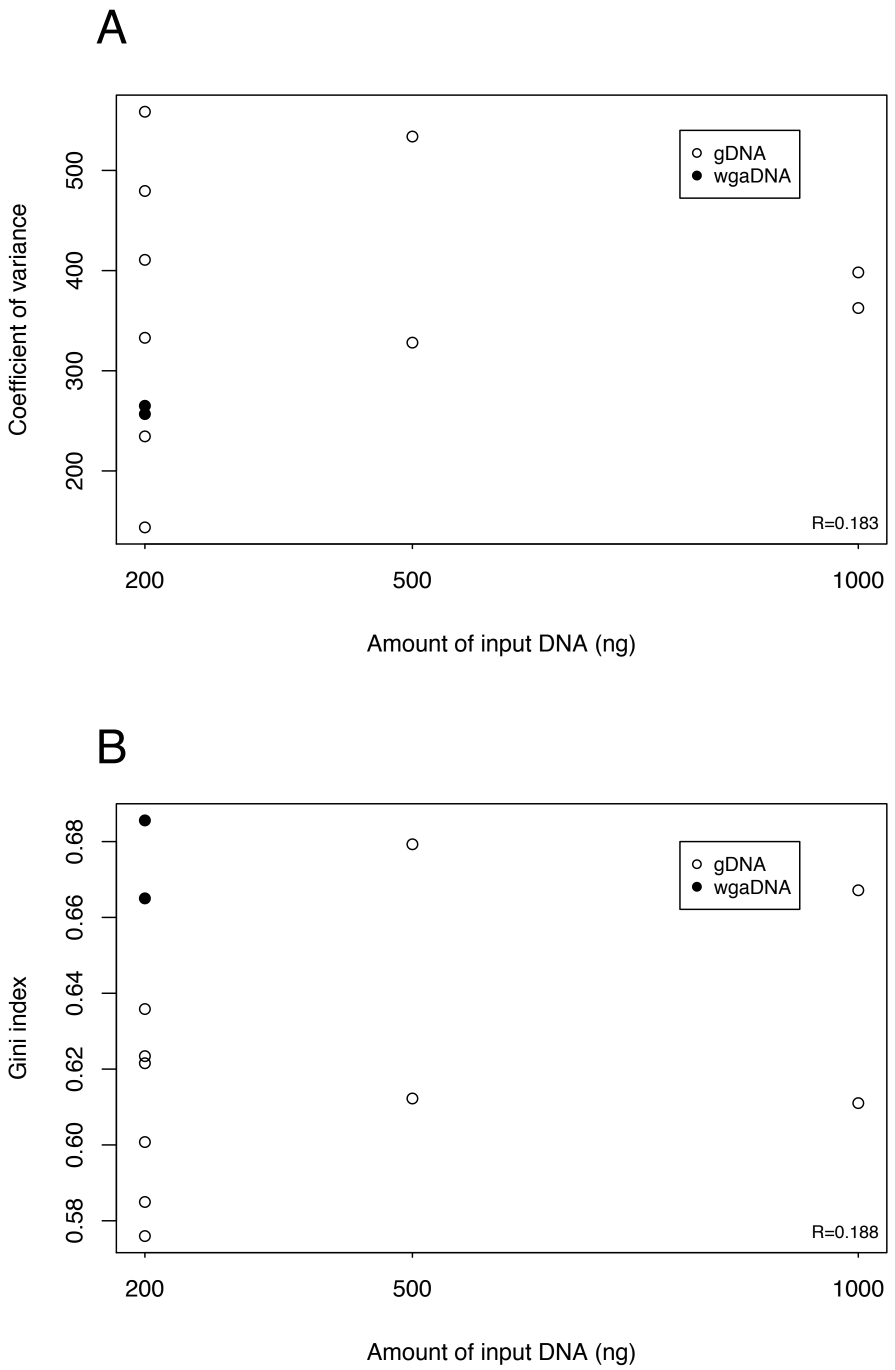

Figure S3. Evenness of sequence coverage in experiments with different amounts of input DNA. Comparison of the evenness of the sequence coverage in experiments with different amounts of input DNA as estimated by A) the coefficient of variation (CV) and B) the Gini index. The CV is the ratio of the standard deviation and the mean in a population. A high CV indicates high variability. The mathematically more complex Gini index measures the inequality among values of a frequency distribution. A Gini index of 0 indicates perfect equality and an index of 1 indicates maximal inequality. Thus, an experiment with uneven coverage is expected to have a high CV and a high Gini index. Both the CV and the Gini index indicate that there is no correlation between the amount of input DNA and the evenness of the coverage.

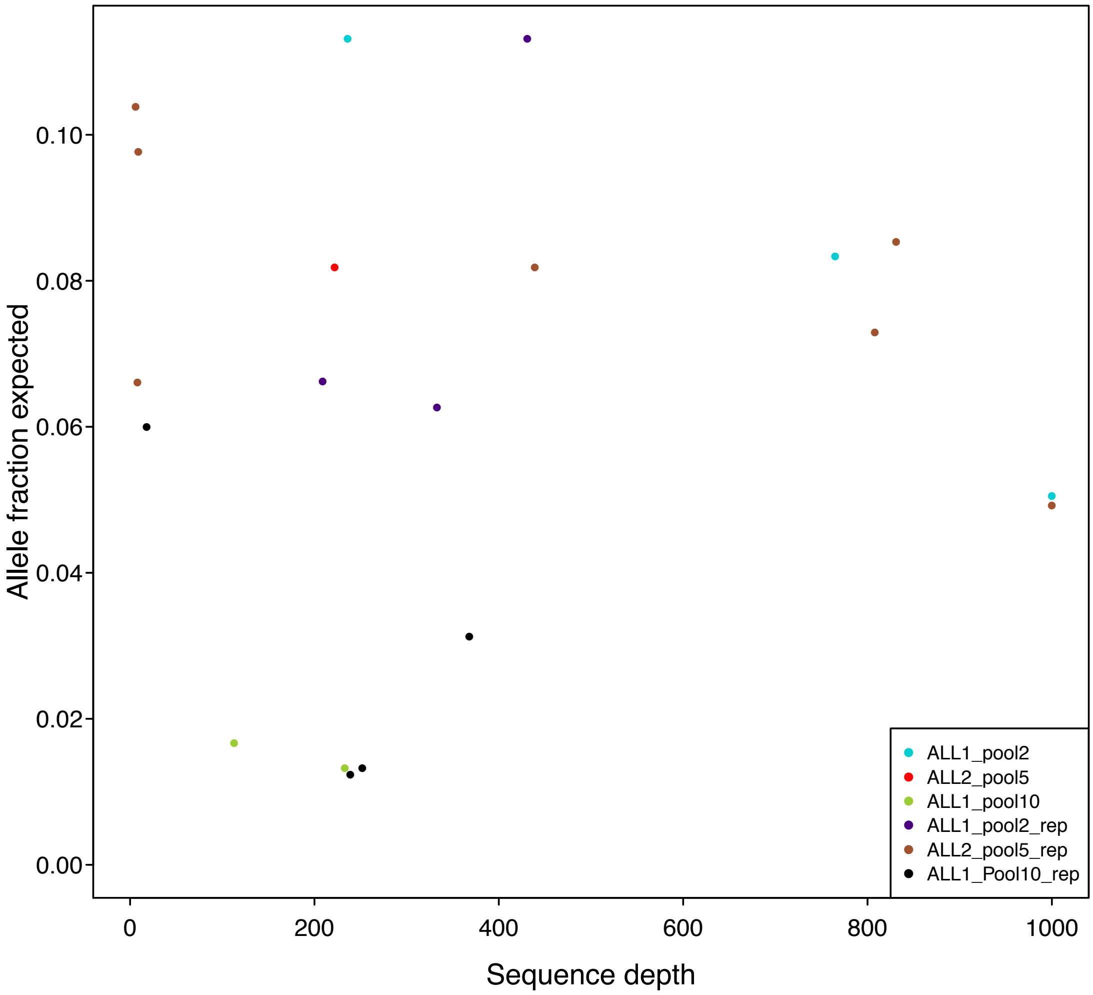

Figure S4. Sequence depth and expected allele fraction of somatic single nucleotide variants (SNVs) that remained undetected in pools. The x-axis shows the sequence depth at positions where a somatic SNV remained undetected in a pool, and the y-axis shows the expected allele fraction of the SNV in that pool. The expected allele fraction is calculated by dividing the allele fraction observed in the individual gDNA experiment with the number of samples in the pool. If the same SNV remained undetected in several pools it is plotted once for each pool. Most SNVs that were undetected in pools had a low sequence depth and a low expected allele fraction. For increased resolution at lower depths, SNVs with a depth >1000 are shown at 1000.

Table S1. Clinical characteristics of ALL samples used in the study

ID	Immunophenotype[a]	%blasts[b]
ALL1	BCP-ALL	90
ALL2	T-ALL	95
ALL3	T-ALL	95
ALL4	T-ALL	95
ALL5	T-ALL	95
ALL6	BCP-ALL	90
ALL7	BCP-ALL	80
ALL8	T-ALL	95
ALL9	BCP-ALL	90
ALL10	BCP-ALL	95
ALL11	BCP-ALL	90
ALL12	BCP-ALL	95
ALL13	BCP-ALL	90
ALL14	BCP-ALL	85
ALL15	BCP-ALL	80
ALL16	BCP-ALL	NA

[a] BCP-ALL: B-cell precursor ALL; T-ALL: T-cell ALL
[b] Estimated percentage of leukemic cells in the sample.
