# Supplementary material for: Accurate detection of subclonal single nucleotide variants in whole genome amplified and pooled cancer samples using HaloPlex target enrichment
Source: BMC Genomics. 2013 Dec 5;14(1):856. doi: 10.1186/1471-2164-14-856 (PMC4046713; doi:10.1186/1471-2164-14-856)
Supplement: Supplementary file 2 — Additional file 2: List of revisions of the HaloPlex target capture protocol between our experiment and the newest version of the protocol. (PDF 405 KB) [file 12864_2013_5568_MOESM2_ESM.pdf]

Recent updates to the HaloPlex protocol

What's New in Version B

- Updates to enrichment validation and quantitation protocol
- Update to kit contents table
- Minor updates to sample mixing and handling instructions

What's New in Version C

- Updated PCR amplification protocol using Herculase II Fusion DNA polymerase
- Support for 501 kb to 5 Mb probe designs, including updated hybridization protocol and kit information
- Support for 16-reaction runs
- Addition of step for mixing the SSC Buffer bead suspension

What's New in Version D

- Updated hybridization protocol including increased hybridization temperature and removal of terminal hold step
- Updated DNA capture protocol
- Updated purification protocol for amplified, enriched DNA
- Updated instructions for validation of restriction digest reactions using the Bioanalyzer system

What's New in Version D.1

- Updated post-enrichment validation analysis recommendations
- Updated sequencing setup advice for the MiSeq platform
- Updated thermal cycler specifications

What's New in Version D.3

- Updated instructions for determination of hybridization time by referral to Box 1 Certificate of Analysis
- Updated instructions for use of Enzyme Strips 1 and 2
- Support for 12-reaction run setup
- Update to enriched library purification protocol

What's New in Version D.4

- Support for HaloPlex Cancer Research Panel designs
- Updated Custom Kit ordering information
- Updated supplier information for NaOH and acetic acid and updated preparation instructions for NaOH
- New Run Time Considerations section
- Support for FFPE-derived DNA samples
- Updated instructions for preparation of RE Master Mix Strip
- Instructions for obtaining Agilent's SureCall analysis software

What's New in Version D.5

- Support for HaloPlex Cardiomyopathy Research Panel designs
